# Genome wide association scan for chronic periodontitis implicates novel locus

**DOI:** 10.1186/1472-6831-14-84

**Published:** 2014-07-09

**Authors:** Ping Feng, Xiaojing Wang, Priscila L Casado, Erika C Küchler, Kathleen Deeley, Jacqueline Noel, Hyongsup Kimm, Ji-Hye Kim, Alex N Haas, Valquiria Quinelato, Leticia L Bonato, Jose M Granjeiro, Cristiano Susin, Alexandre R Vieira

**Affiliations:** 1 Department of Oral Biology, School of Dental Medicine, University of Pittsburgh, 3501 Terrace Street, Pittsburgh, PA 15261, USA; 2 Department of Pediatric Dentistry, School of Dental Medicine, University of Pittsburgh, Pittsburgh, PA, USA; 3 Guiyang Stomatological Hospital, Guiyang, Guizhou, China; 4 Research Division, Orthopedics and Traumatology National Institute, Rio de Janeiro, RJ, Brazil; 5 Clinical Research Unit and Biology Institute, Federal Fluminense University, Niterói, RJ, Brazil; 6 Department of Periodontology, Faculty of Dentistry, Federal University of Rio Grande do Sul, Porto Alegre, RS, Brazil; 7 National Institute of Metrology, Quality and Technology, Rio de Janeiro, RJ, Brazil; 8 Department of Periodontics, Georgia Regents University College of Dental Medicine, Augusta, GA, USA

**Keywords:** Periodontitis, Chronic periodontitis, Aggressive periodontitis, Genetics, Medical genetics, Genetic polymorphism

## Abstract

**Background:**

There is evidence for a genetic contribution to chronic periodontitis. In this study, we conducted a genome wide association study among 866 participants of the University of Pittsburgh Dental Registry and DNA Repository, whose periodontal diagnosis ranged from healthy (N = 767) to severe chronic periodontitis (N = 99).

**Methods:**

Genotyping^i^ of over half-million single nucleotide polymorphisms was determined. Analyses were done twice, first in the complete dataset of all ethnicities, and second including only samples defined as self-reported Whites. From the top 100 results, twenty single nucleotide polymorphisms had consistent results in both analyses (borderline p-values ranging from 1E-05 to 1E-6) and were selected to be tested in two independent datasets derived from 1,460 individuals from Porto Alegre, and 359 from Rio de Janeiro, Brazil. Meta-analyses of the Single nucleotide polymorphisms showing a trend for association in the independent dataset were performed.

**Results:**

The rs1477403 marker located on 16q22.3 showed suggestive association in the discovery phase and in the Porto Alegre dataset (p = 0.05). The meta-analysis suggested the less common allele decreases the risk of chronic periodontitis.

**Conclusions:**

Our data offer a clear hypothesis to be independently tested regarding the contribution of the 16q22.3 locus to chronic periodontitis.

## Background

Although family studies suggest that environmental factors are the major determinants of variance in chronic periodontitis [1-5], comparisons between reared-together and -apart adult monozygous twins indicate that early family environment has no appreciable influence on periodontal status of adults [6]. Several association studies have been published over the last decade aiming to identify genetic factors contributing to chronic periodontitis [7]; however, the results are not necessarily the same depending on the population studied [8].

More recently, a genome wide association study [9,10] including 1,020 and 4,504 participants self-defined as Whites selected from the Atherosclerosis Risk in Communities (ARIC) longitudinal cohort suggested a few novel loci to be possible contributors to chronic periodontitis although none of them reached formal statistical significance. Additionally, the two lists of associated single nucleotide polymorphisms from the ARIC studied samples [9,10] did not obviously overlap. Divaris et al. [9] also included analyses based on bacterial colonization of eight species and the results suggested additional loci that may contribute to individual susceptibility of being colonized by specific bacterial groups.

In this study, we took into consideration the presence of ethnic admixture to investigate the association between genetic variation and chronic periodontitis. A genome-wide association scan for chronic periodontitis was conducted, including analysis adjusted by smoking habits and diabetes status and staged by incrementally adding samples from different ethnicities, to address the role of genes in this disease. Our results offer a clear hypothesis to be independently tested regarding the contribution to the 16q22.3 and 21q22.11 loci to chronic periodontitis.

## Methods

### Discovery sample

All patients were participants in the Dental Registry and DNA Repository (DRDR) of the University of Pittsburgh School of Dental Medicine. Starting in September of 2006, all individuals that seek treatment at the University of Pittsburgh School of Dental Medicine have been invited to be part of the registry. They give written informed consent authorizing the extraction of information from their dental records. Also, they provide a saliva sample from which DNA can be extracted. Unstimulated saliva samples were obtained from all participants (individuals were asked to spit) and stored^ii^ at room temperature until being processed. No centrifugation was performed in the saliva samples. DNA was extracted according to the manufacturer’s instructions. The University of Pittsburgh Institutional Review Board approves this project and all individuals signed a written informed consent document prior to participation.

In January 2010, data from 886 individuals were extracted from the registry for this project. Twenty individuals younger than 17 years of age were excluded. The mean age of participants was 41.2 years with ages ranging from 25 to 89 years. One hundred and twenty-eight were smokers and 44 had diabetes. Participants with 30% or more teeth with sites of clinical attachment loss of five millimeters or more were defined as having chronic periodontitis [11,12]. Participants who had attachment loss of five millimeters in less than 30% of sites were not included in the study. No cases that were diagnosed as aggressive periodontitis were included in this study. Ninety-nine patients were diagnosed with chronic periodontitis (42 women and 57 men with ages ranging from 30 to 83 years). Based on their self-reported ethnicity 63 were White, 32 Black, and four belonged to other ethnicities and these patients comprised the affected group. Non-affected individuals were 767 (382 women and 385 men with ages ranging from 25 to 84 years of age). Based on their self-reported ethnicity 543 declare being White, 158 Black, and 66 pertaining to other groups (Table 1). Additional details are presented as Additional file 1 (Appendix 1: “Phenotype Definition”). The Dental Registry and DNA Repository is a hospital-based project and it is expected that clinical data will be recorded by a number of different professionals. All research records were reviewed to exclude the possibility that cases may have aggressive periodontitis.

**Table 1:** Summary of the study populations

**Variable**		**Pittsburgh**	**Porto Alegre**	**Rio de Janeiro**
	N	866	1,460	359
Periodontitis status	Affected	99 (11.4%)	430 (29.4%)	183 (51%)
	Non-Affected	767 (88.6%)	1,030 (70.6%)	176 (49%)
Mean age (Years)		41	40	56
Sex	Females	439 (50.7%)	784 (53.7%)	257 (71.6%)
	Males	427 (49.3%)	676 (46.3%)	105 (28.4%)
Ethnic background	White	606 (70%)	1,188 (81.4%)	288 (80.2%)
	Black	190 (21.9%)	272 (18.6%)	71 (19.8%)
	Other	70 (8.1%)	0	0
Ethnic background	White	63	348	147
In individuals with	Black	32	82	36
With Periodontitis	Other	4	0	0
Smoker		128 (14.8%)	430 (29.4%)	39 (10.9%)
Self-reported diabetes		44 (5.1%)	40 (2.7%)	31 (8.6%)

DNA samples were genotyped for 620,901 single nucleotide polymorphisms (SNPs)^iii^. Details of our power calculations are presented as Additional file 1 (Appendix 2: “Power Calculations of the Discovery Sample” and Additional file 1). The particular SNP array chosen includes SNPs that are representative for individuals of both African and European ancestry [13], which we considered an important aspect of the design, since the study group was comprised of individuals that are self-reported Whites or Blacks.

Association between periodontitis affection status and each single nucleotide polymorphism across the whole genome was tested using PLINK [14] and all analyses were adjusted for age, sex, diabetes status (yes or no), and smoking status (smoker or non-smoker), variables that are associated with distinct periodontal disease levels [15-19]. Data on ex-smokers was not consistent in all registry dataset and this variable was not used in the analysis. In the analysis of the complete dataset, we also adjusted for the principal components from an evaluation of population structure as described in the Additional file 1 (Appendix 3: ”Genome Wide Analysis,” Appendix 4: “Adjustment for Ethnicity in the genome Wide Analysis,” Additional file 1). We then repeated these analyses with samples from White individuals only (Additional file 1). To account for multiple testing, a p-value lower than 1E-07 (0.05/473,514) was considered statistically significant.

### Follow up samples

From the 100 results with the lowest p-values, we selected the most consistent findings (the findings that continued to show a trend of association) of both analyses (*i.e*., analyses of the complete dataset and with samples from White individuals only; Additional file 1), which comprised of twenty single nucleotide polymorphisms (Table 2). These were then tested in two independent population-based cohorts from Brazil. Details of these samples are provided in Table 1 and as Additional file 1 (Appendix 5: “Details of the Follow Up Samples”). Definition of periodontal disease used was the same as described in the discovery sample.

**Table 2:** Summary of the results of the genome wide association scans and independent analysis for chronic periodontitis

**Marker**	**Minor allele frequency**	**Locus**	**Location/gene**	**Genome wide scan p-value all samples (99 affected, 767 unaffected)**	**Genome wide scan p-value whites only (63 affected, 543 unaffected)**	**Independent Porto Alegre cohort p-value (430 affected, 1,030 unaffected)**	**Independent Rio de Janeiro cohort p-value (183 affected, 176 unaffected)**
rs10858049	0.19	1p13.2	*DENND2C*	9.54E-05	0.01	0.78	0.3
rs4572866	0.19	4p15.2	Intergenic	5.80E-05	0.0006	0.49	0.42
rs6905786	0.45	6q16.2	Intergenic	1.41E-05	0.0003	0.65	0.72
rs7740539	0.26	6q21	Intergenic	1.39E-05	2.85E-05	0.16	0.65
rs10266202	0.41	7p21.3	Intergenic	3.89E-05	0.0003	0.68	0.4
rs4735081	0.4	8q23.1	Intergenic	1.80E-05	4.71E-05	0.48	0.86
rs6597536	0.15	9q34.13	*MED27*	3.62E-05	0.0002	0.91	0.9
rs1954179	0.34	10q25.2	*RBM20*	7.42E-05	0.0008	0.85	0.82
rs10501568	0.1	11p14.1	*DLG2*	8.81E-06	9.38E-05	0.58	0.66
rs1026477	0.47	11p14.1	*MPPED2*	5.48E-05	2.79E-06	0.68	0.98
rs982322	0.31	12q14.1	*SLC16A7*	3.99E-05	0.0008	0.48	0.74
rs1913208	0.42	13q33.1	Intergenic	6.46E-05	0.0004	0.17	0.6
rs1477403	0.46	16q22.3	Intergenic	6.18E-05	0.0001	0.05	0.66
rs1558878	0.45	17q24.2	*ARSG*	8.53E-05	0.0004	0.63	0.29
rs8066940	0.42	17q25.3	Intergenic	2.79E-05	0.0004	0.37	0.3
rs7254232	0.38	19q13.32	Intergenic	2.57E-06	0.0005	0.14	0.2
rs2833017	0.45	21q22.11	Intergenic	5.20E-05	0.0004	0.32	0.001
rs9305434	0.33	21q22.11	Intergenic	7.91E-05	0.0008	0.41	0.008
rs2048126	0.46	21q22.11	Intergenic	8.43E-05	0.0008	0.43	0.001
rs466092	0.25	21q22.3	*MX2*	1.28E-05	1.10E-05	0.14	0.76

Note: P-values are related to the differences in distribution of single nucleotide polymorphisms between affected and unaffected individuals.

For the 20 single nucleotide polymorphisms selected to this independent test, genotyping was carried out using TaqMan chemistry [20] and end-point analysis.^iv^ All genetic markers were in Hardy-Weinberg equilibrium (data not shown). To determine the association between the disease and any allele or genotype frequency, we used logistic regression adjusted for age, sex, ethnicity, diabetes status, and smoking status using PLINK [14]. Data on ex-smokers was not available in these datasets. The sample from Porto Alegre was also adjusted by body mass index as well since these data were available and this variable has been associated with periodontal diseases. P-values equal or lower than 0.0025 (0.05/20) were considered statistically significant for the follow up study results.

### Meta-analysis

In order to derive a summary statistic for association with the four SNPs that showed a trend for association in either of the follow up studied samples from Brazil, a random-effects meta analysis model was used to estimate the odds ratio for the presence of the associated allele determined by the genome-wide association analysis. Before pooling the data, we estimated Cochran’s Q statistic, which indicates the degree of heterogeneity. There was no significant evidence of heterogeneity overall (Q = 2.7, p = 0.264). A random-effects model was used because it includes components of variance both within and between studies. Moreover, because it generally yields a wider confidence interval than a fixed-effects model, the random-effects mode is more conservative [21]. The complete dataset from Pittsburgh was used. MedCalc version 13 was used (MedCalc Software, Ostend, Belgium).

## Results

### Genome wide associations study

**Figure 1:**
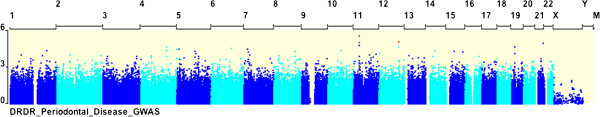
**Manhattan plot summarizing the results of the genome wide scan study.** No p-values reached the threshold of significance of 1E-07. Intercalating blue colors indicate different chromosomes. The Y chromosome was not analyzed, hence no markers (blue color) appear. Red dots indicate the most significant results.

Quality filters on single nucleotide polymorphisms were applied before analysis including exclusion of monomorphic and high missing rate single nucleotide polymorphisms (>10%). From the total 620,901 single nucleotide polymorphisms in the chip, 477,410 markers passed quality control. There were an additional 3,896 markers that were not in Hardy-Weinberg equilibrium (p ≤ 0.0001) or minor allele frequencies were not informative (frequency ≤ 0.05) and were also excluded. A total of 473,514 single nucleotide polymorphism markers were used for analysis. Although no single nucleotide polymorphisms met conservative criteria for genome-wide significance, multiple suggestive loci, represented by one or more associated markers with p-values of between 1E-5 and 1E-6, were observed in the discovery sample (Figure 1).

### Follow up studies

We selected 20 single nucleotide polymorphisms that had consistent results in both analyses of the total sample and self-reported Whites only to test in two independent cohorts (Table 2). Inclusion of sex, ethnicity, diabetes status, smoking habits, and body mass index along with age in the model did not substantially change the results and data presented here are based on the simplest model adjusted only by age. The rs1477403 marker located on 16q22.3 was the only one that showed a trend for association in the cohort from Porto Alegre, Brazil [odds ratio = 1.2 (95% confidence interval 1.0-1.47); p = 0.05 for the allele distribution, Table 2]. Three markers in 21q22.11 showed a trend for association (nominal p-values lower than 0.05) with chronic periodontitis in the cohort from Rio de Janeiro (Table 2). These markers are in strong linkage disequilibrium with each other (D’ = 1.0).

### Meta-analysis

**Figure 2:**
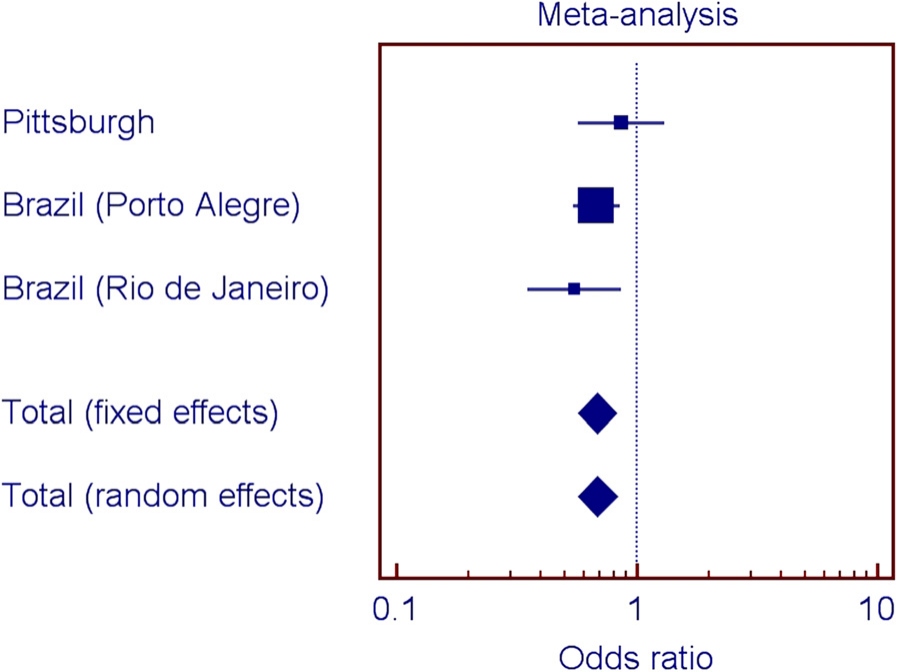
Summary of meta-analysis results for rs1477403 (16q22.3).

**Figure 3:**
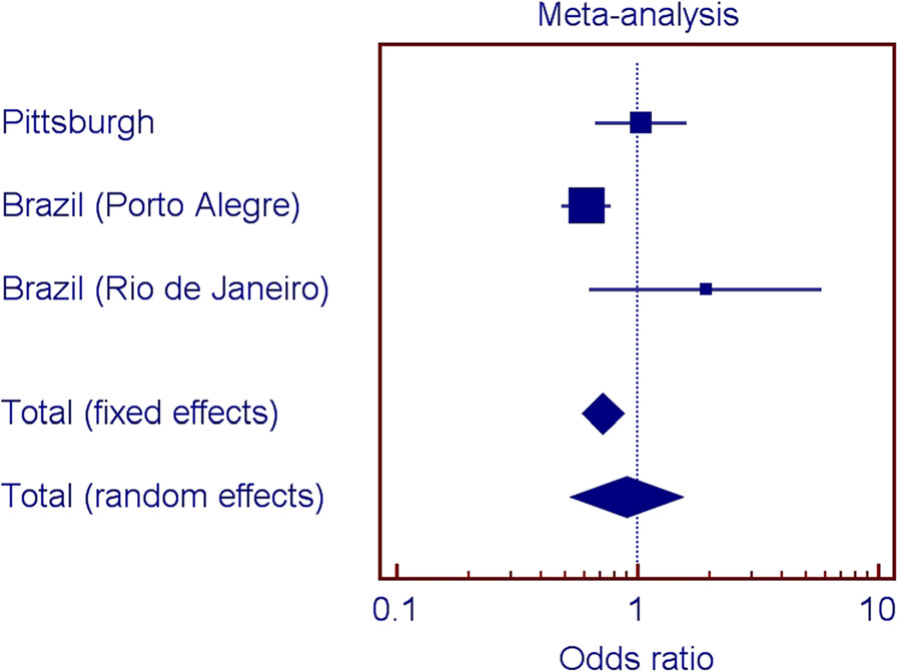
Summary of meta-analysis results for rs9305434 (21q22.11).

**Figure 4:**
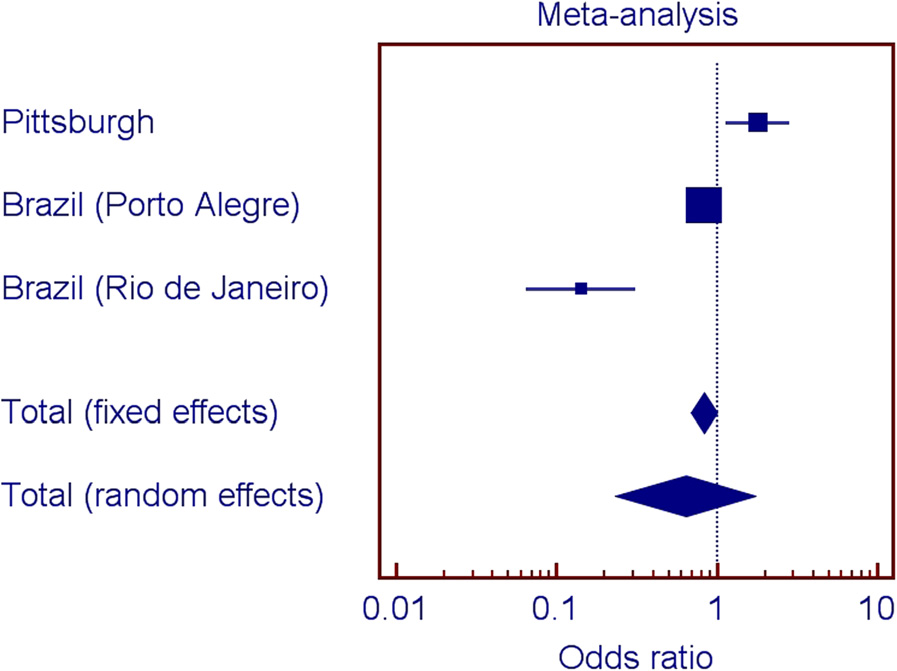
Summary of meta-analysis results for rs2833017 (21q22.11).

**Figure 5:**
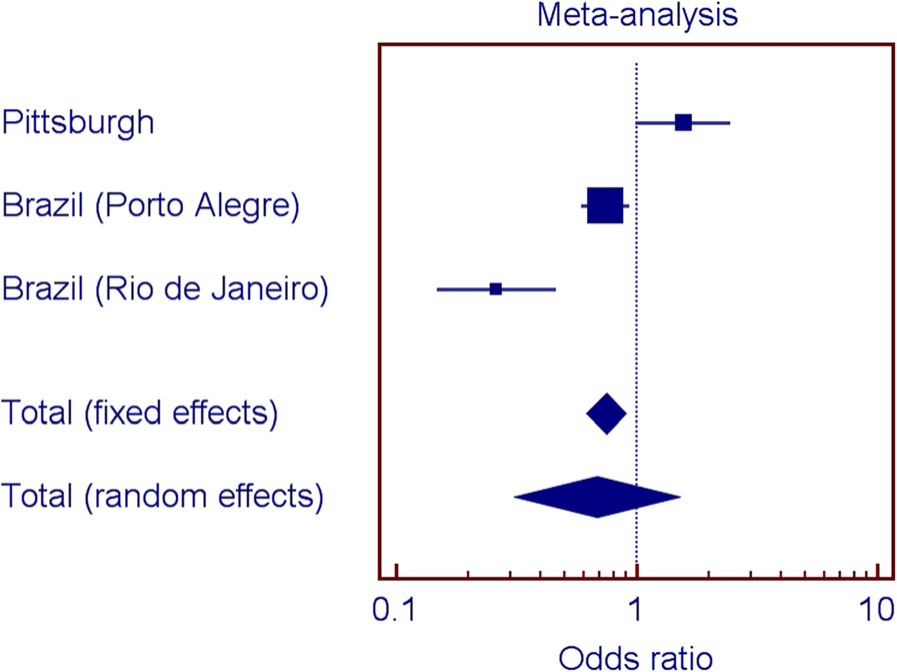
Summary of meta-analysis results for rs2048126 (21q22.11).

Figures 2, 3, 4, and 5 show the odds ratios for the association of rs1477403 in 16q22.3 and the three SNPs in 21q22.11 in the samples from Pittsburgh, and the two Brazilian cohorts. Only rs1477403 show an association that is consistent in direction for the three studied populations. The less common allele is under-represented in individuals affected by chronic periodontitis, suggesting a protective effect. The inconsistent results across the studies for the other three 21q22.11 markers suggest a true association between chronic periodontitis and the locus probably does not exist.

## Discussion

In this study, 2,685 DNA samples were analyzed coming from two cohort studies and one case–control dataset. These different study designs explain the variation of periodontal disease frequency in each of the study groups ranging from 11% to 50%.

The first step of our study included a genome-wide analysis. Chronic periodontitis has a prevalence of over 47% in the United States based on NHANES data [22], and in general lower sample sizes are necessary to study a common disease than a rare disease [23]. However, with the relatively modest number of affected individuals and anticipated statistical power, we implemented two strategies to improve statistical power. We included at least four controls for each case, which is considered the golden standard for the numbers of cases and controls to be collected in a case–control genetics study [23]. The other approaching was to use cases with at least 30% sites of the mouth affected by chronic periodontitis, hence avoiding the inclusion of less severe cases. This approach is thought to maximize the variance of predictor variables (each genetic variant or X), which according to b_x_ ± t_n-m-1_;α√MSE/nV_x_(1-R^2^) where MSE is the mean square error, n is the sample size, V_x_ is the variance of X, and (1-R^2^) is the proportion of the variance X not shared by any other variables in the model, will increase power and precision [24]. While no single nucleotide polymorphism exhibited association at genome wide significance, several genomic regions showed suggestive evidence for association. However, only four genetic markers in two loci showed also a trend for association in independent experiments with different population datasets, and only one marker showed association when the samples were pulled. These results are interesting because one experiment was done in a hospital-based cohort which clinical data is obtained from different professionals and greater heterogeneity, and the following experiments were done in population-based cohorts and data were collected with experimental rigor to increase homogeneity. rs1477403 is located at 16q22.3 and in a sequence of an uncharacterized non-coding RNA (LOC100506172). The nucleotide change is not conserved in mouse, chimp, orangutan, or macaque according to the data available at UCSC Genome Browser and is unlikely to have a direct functional role, but this possibility cannot be excluded.

Our approach to select markers to follow up included comparing the top 100 results of the two genome wide scan analyses. We could have prioritized markers based on our initial power calculations. However, a fair assumption for periodontal diseases is that individual gene contributions are small and if we used odds ratio cut-offs lower than 1.5, we would likely have several hundred if not thousand possible markers to follow. Two-stage designs for manipulating ranked SNPs based on p-values have been shown to improve the rankings and to decrease overestimated significance values [25-28].We also performed a met-analysis to help interpret the results of the analyses of the four SNPs in the three population groups. If one population produces a large p-value for a given SNP when two other populations produced small ones for that SNP, it seems there are several possible reasons. One would be that the SNP is truly associated with the trait in the populations conferring the signal, but the SNP is not associated with the trait in the third population. Another possibility is that the SNP is associated with the trait in all populations, but the sample of individuals collected from one of the populations by chance happened to provide low power. A third possibility of course is that the SNP is not associated with the trait, and the two populations that showed a signal were both false positives. If there is, in truth, association in the third population, but the sample happened to display low power, then while the direction of any effect seen in the sample would be expected to be the same, it also seems not unlikely that by chance it might actually be opposite (low p-values mean effect sizes near zero in a given sample, and as such, the “effect” could be in either direction). We hypothesize that the signal for the 16q22.3 SNP is real, despite the individual analysis of one of the Brazilian populations does not indicate association (Figure 2). On the other hand, since the signals for the SNPs in 21q22.11 are not consistent (Figures 2 through 4), we hypothesize the evidence for association with this locus is a false positive. These analyses exemplify the challenge of interpreting results for these kinds of studies.

The first genome wide association study in periodontitis studied the aggressive type of the disease [8]. This study identified an association with a marker in the locus of *GLT6D1* and functional experiments suggested that reduced GATA3 binding affinity to the *GLT6D1* locus could be a component of the pathophysiology of periodontitis [8]. This locus is not one we are suggesting to be associated with chronic periodontitis. The lack of overlap between our findings and of the others [9,10] in genome wide scanning for chronic periodontitis and the study of Schäfer et al. [8] is likely due to the fact that these two conditions have distinct genetic influences. We have previously shown that aggressive periodontitis aggregates in families and its most parsimonious mode of inheritance is a semi-general transmission model that allows the heterozygote transmission to vary [29]. This is very distinct from what we see in chronic periodontitis in which no clear familial aggregation can be detected.

Our study benefits from several strengths including genome-wide single nucleotide polymorphism data and rigorous and thorough assessment of phenotypes. Genotyping and quality control/quality assurance yielded data of exceptional quality. Moreover, as one of the first genome wide association studies for chronic periodontitis reported to date, this study accomplished the principal goal (of the non-hypothesis-based genome wide association study design), of generating interest in genes and genomic regions previously unstudied in the context of oral health. However this study also highlights the challenges of identifying genes involved in common complex disease, namely, that numerous genes, mostly of small effect sizes, are likely to contribute to periodontitis, and that discovery of individual variants may be exceedingly difficult. Our study populations had a mix of individuals of both White and Black heritage and this further complicates any analysis since allele frequency may be disparate between different populations. Even though we carefully took into consideration this factor, we cannot exclude the possibility that the suggestive associations we found are influenced by variation in ethnic background of the samples. While research into the genetics of periodontitis lags behind many other prominent common complex diseases, this study provides a launching pad for future candidate gene and functional studies of periodontal diseases. The public availability of these data via online portals will facilitate the utility of this study in designing future efforts and cross-study collaborations to understand the genetics of periodontal diseases.

## Conclusions

Our data offer a clear hypothesis to be independently tested regarding the contribution of the 16q22.3 locus to chronic periodontitis.

## Endnotes

^i^Performed in a Illumina 610-Quad platform.

^ii^Stored in Oragene DNA Self-Collection kits (DNA Genotek Inc., Ottawa, ON, Canada).

^iii^Using the Illumina Human610-Quadv1_B BeadChip (Illumina Inc., San Diego, CA, USA).

^iv^Performed on an Applied Biosystems 7900 HT Sequence Detection System machine (Applied Biosystems Inc., Foster City, CA, USA).

## Competing interests

The authors have no competing interests to declare.

## Authors’ contributions

PF, PLC, KD, HK J-HK performed DNA manipulation and all laboratory experiments. PF, XW, PLC, ECK, and ARV analyzed and interpreted the data. PLC, JN, ANH, VQ, LLB, CS, JMG, and ARV performed activities related to subject recruitment, phenotype definitions, and biological sample collection. PLC, JMG, CS, and ARV designed the original cohort studies. All authors critically revised the manuscript. All authors read and approved the final manuscript.

## Pre-publication history

The pre-publication history for this paper can be accessed here:

http://www.biomedcentral.com/1472-6831/14/84/prepub

## Supplementary Material

Additional file 1 Additional information on phenotype definition, power calculations and genome wide association analyses. Click here for file
