# Supplementary material for: Genome wide association scan for chronic periodontitis implicates novel locus
Source: BMC Oral Health. 2014 Jul 9;14:84. doi: 10.1186/1472-6831-14-84 (PMC4096424; doi:10.1186/1472-6831-14-84)

**Additional files**

**Appendix 1: Phenotype Definition**

In the three studied groups, subjects with 30% or more teeth with sites of clinical attachment loss of five millimeters or more were defined as having chronic periodontitis.^1,2^

The periodontal exam information that was part of the University of Pittsburgh School of Dental Medicine Dental Registry and DNA Repository records was obtained from multiple examiners, which could be dental students, residents, or faculty practitioners. However, the examiners follow the same general instructions in regards to obtaining these assessments. Despite not being possible to generate intra- and inter-examiner, the quality of the data is commensurate to other similar studies where information is obtained from existing records.

The samples from Porto Alegre, Brazil had their clinical examinations were performed in a mobile examination center consisting of a trailer equipped with a complete dental unit. Four dentists conducted the clinical examinations, and two trained dental assistants recorded the data on prepared record sheets. A full-mouth clinical examination, excluding third molars, was performed. The examination included an assessment of the status of the permanent teeth and periodontal tissue. The examiners were calibrated at two time points: before, and three months following the start of the study. In addition, the examiners’ reproducibility in assessing tooth loss, caries experience, and attachment loss was assessed during the fieldwork. One examiner with the most clinical experience (C.S.) served as the “gold standard” examiner. A total of 57 subjects, divided into four groups ranging from eight to twenty subjects, were used for the reproducibility assessment. In one of the groups, the replicate measurements consisted of repeated measurements by the reference examiner. In each of the remaining three groups, one examiner and the reference examiner made the replicate measurements. The reproducibility of measurements was assessed by the intraclass correlation coefficient and the kappa statistics. The intraclass correlation coefficients for the number of missing teeth per subject ranged between 0.99 and 1.0, and the kappa coefficients for the type of missing teeth ranged between 0.98 and 1.0. The kappa coefficients for caries experience ranged from 0.89 to 0.98. The intraclass correlation coefficients for the percentage of teeth with attachment loss ≥ 5 millimeters ranged between 0.82 and 0.97.

The samples from Rio de Janeiro, Brazil were collected by two calibrated examiners (V.Q. and L.L.B.) trained by an experienced dentist (P.L.C.). Exams were performed in a fully equipped dental office. The intra-examiner agreement was assessed by a second clinical exam in ten subjects after two weeks, with a kappa of 0.99. Cohen’s kappa values for agreement between examiners were 0.91.

**Appendix 2: Power Calculations of the Discovery Sample**

It is difficult to calculate power exactly for such a complex and multi-faceted project, and as discussed above we plan to integrate results from the different data sources both qualitatively and quantitatively, but the following table gives a rough sense of the power of our samples. We estimated the power as a function of the genotype relative risk under a multiplicative model using Purcell’s Genetic Power Calculator (http://pngu.mgh.harvard.edu/~purcell/gpc/) for a simple chi-squared test of allelic association assuming an r^2^ of 0.8 between the marker and trait loci. We assumed a population prevalence of 10%, but prevalence numbers of up to 30% give essentially identical results. Supplemental Table 1 uses an extremely conservative genomic-wide p-value cutoff of 10^-7^, and shows that we will have power to detect relative risks of 1.8. Moreover, since we are not interested in the power to detect a single specific locus per se but rather the power to detect some subset of all relevant loci, the real power may be potentially much higher than the estimates reported here. This means that we are aware our sample cannot detect markers with relative risks below 1.5. Hence, we may be able to detect markers with higher relative risks if they exist for periodontal diseases.

Supplemental Table 1: Summary of power calculations.

|  | **Relative Risk** |  |  |  |
| --- | --- | --- | --- | --- |
|  | **1.2** | **1.3** | **1.5** | **1.8** |
| **N = 886**  **(Dental Registry and DNA Repository)** | 0 | 0 | 12% | 88% |

**Appendix 3:Genome Wide Analysis**

Since this dataset was comprised by DNA from individuals that self-identified themselves either as Whites or as Blacks (with some few exceptions), we decided to evaluate the correlation between self-reported ethnicity and genotypes. Extremely high concordance rates were demonstrated by principal component analysis using independent markers across the genome between self-reported ethnicity and genotypes (>98%). Since the distribution of self-reported ethnicity between affected and non-affected individuals was different, we analyzed the data in two steps: first, we performed the genome wide association scan analysis on the complete dataset with all self-reported ethnicities with adjustment for the first five principal components given that the first two (or any pair of two) components cannot completely distinguish Whites from non-Whites samples. We repeated this step by only adjusting for the first two principal components but the results remained almost unchanged. Based on these results, we performed genome wide association regression analysis also modeling for all five principal components. We did not find lack of collinearity (high correlations among any principal component; data not shown) and we considered appropriate to adjust all of the principal components in the regression analysis. Second, we limited analysis to self-reported Whites only.

**Appendix 4: Adjustment for Ethnicity in the Genome Wide Analysis**

A first round of principal component analysis (PCA) was conducted using all autosomal single nucleotide polymorphisms (SNPs) with minor allele frequency >0.01. PCA was performed using the computer program EIGENSOFT 2.0.^3,4^ SNP loadings for the leading components were compared with a normal distribution to determine whether these components depended on many SNPs across the genome or if they were dominated by relatively few SNPs all mapped to a few small chromosome regions with extended linkage disequilibrium (LD), as would be expected when the given component reflected population structure or a more localized LD effect, respectively. To correct for the local effects, the PCA was re-applied in a reduced SNP set. In this reduced SNP set, i) SNPs with loadings that deviated from their expected normal quantiles with a distance greater than one were excluded along all leading components; ii) remaining SNPs were pruned using the "in-depth-pairwise" option in PLINK 1.03^5^ such that all SNPs within a given window size of 100 had pairwise *r*^2^< 0.2; iii) each SNP was regressed on the previous two SNPs, and the residual entered into the PCA. SNP loadings on all components deemed significant by the Tracy-Widom statistic were re-inspected to make sure that no component was dominated by a small LD region of the genome. In case there were still leading components dominated by local LD regions, the second round of PCA was repeated with adjusted parameters until no component was dominated by a small LD region.^6^

The best pairwise multidimensional scaling (MDS) plots for the first five components are shown in Supplemental Figure 1. Plots were generated using PLINK1.03.^5^ All SNPs that passed quality control were pruned such that all SNPs within a given window size of 100 had pairwise *r*2 < 0.2. Pairwise IBS distance was calculated using all autosomal SNPs that remained after pruning. Five nearest neighbors were identified for each individual based upon the pairwise IBS distance. IBS distance to each of the five nearest neighbors was then transformed into a *Z* score. Individuals with a minimum *Z* score among the five nearest neighbors less than -4 were excluded from analysis as population outliers. MDS dimensions were extracted using the "MDS-plot" option.


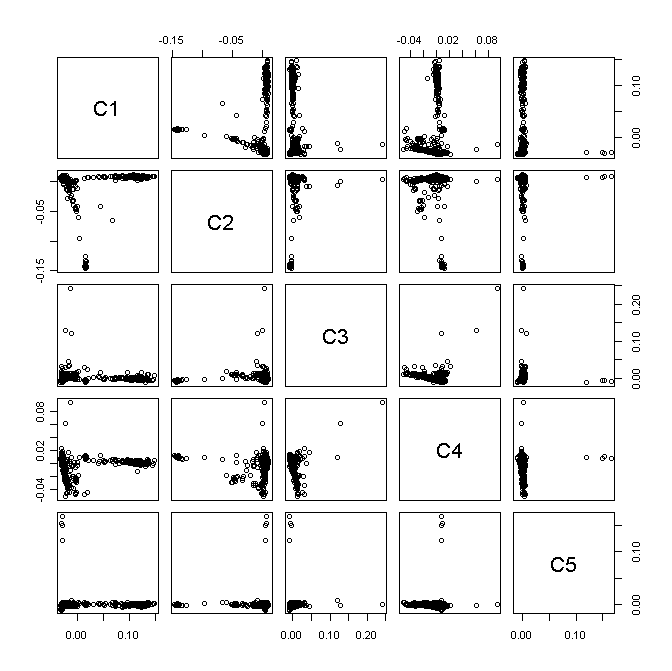


Supplemental Figure 1: The best pairwise multidimensional scaling (MDS) plots for the first five components.

It is a general result of the PCA that the first two PCs separate the data to the maximum extend (analogous for MDS).


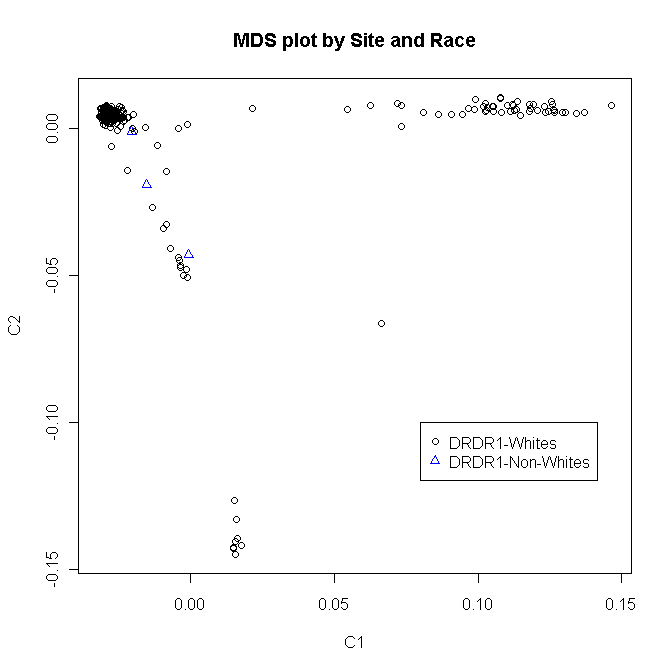

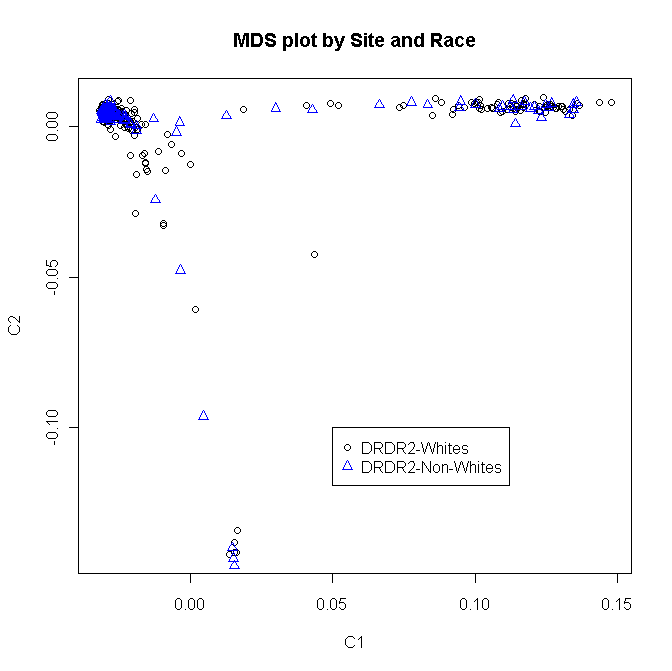


Supplemental Figure 2: MDS plots by site and race.

The sample was divided in two groups based on the timing of genotyping (DRDR1 and DRDR2, MDS plots; Supplemental Figure 2) to evaluate how C1 and C2 discriminated self-report White versus non-White samples. However, as it can be seen, C1 and C2 could not successfully distinguish White/Non-Whites samples, especially in DRDR2.

Thus, we extended this to other pairs (such as C1 versus C3, C3 versus C5 etc.) within the first five components. Unfortunately, none of any pair performs well.

Based on above facts, we decided to adjust in our genome wide association analysis regression model for all five PCs as no multicollinearity (high correlations among these PCs) existed. The pairwise correlation matrix of the five principal components is presented in Supplemental Figure 3.

**C1 C2 C3 C4 C5**

**C1** 1.000000e+00 -2.975268e-08 -4.262633e-08 -2.793244e-08 -5.687919e-08

**C2** -2.975268e-08 1.000000e+00 7.511811e-08 -1.000992e-07 2.456112e-08

**C3** -4.262633e-08 7.511811e-08 1.000000e+00 2.440027e-07 -5.276925e-08

**C4** -2.793244e-08 -1.000992e-07 2.440027e-07 1.000000e+00 6.525500e-08

**C5** -5.687919e-08 2.456112e-08 -5.276925e-08 6.525500e-08 1.000000e+00

Supplemental Figure 3: Pairwise correlation matrix of the five principal components.

Judging from above pairwise correlation matrix, the correlations between any two PCs are very low. These analyses however do not take into account the possible presence of linear combination of the PCs. Despite this limitation, we decided to adjust the analyses by all PCs in our regression model.

**References Cited**

1. Susin C, Dalla Vecchia CF, Oppermann RV, Haugejorden O, Albandar JM. (2004). Periodontal attachment loss in an urban population of Brazilian adults: effect of demographic, behavioral, and environmental risk indicators. *J Periodontol* 2004;75:1033-1041.
2. Susin C, Haas AN, Valle PM, Oppermann RV, Albandar JM. Prevalence and risk indicators for chronic periodontitis in adolescents and young adults in south Brazil. *J Clin Periodontol* 2001;38:326-333.
3. Patterson N, Price AL, Reich D. Population structure and eigenanalysis. *PLoS Genet* 2006;2:e190.
4. Price AL, Patterson NJ, Plenge RM, Weinblatt ME, Shadick NA, Reich D. Principal components analysis corrects for stratification in genome-wide association studies. *Nature Genet* 2006;38:904–909.
5. Purcell S, Neale B, Todd-Brown K, et al. PLINK: a tool set for whole-genome association and population-based linkage analyses. *Ame J Hum Genet* 2007;81:559-575.
6. Wang D, Sun Y, Berlin JA, Wilcox MA, Li Q. Comparison of methods for correcting population stratification in a genome-wide association study of rheumatoid arthritis: principal-component analysis versus multidimensional scaling. *BMC Procedures* 2009;3(Suppl 7):S109.

**Supplemental Figure 4: Quantile-Quantile (Q-Q) Plot Between Analysis of the Total Sample versus just Whites**


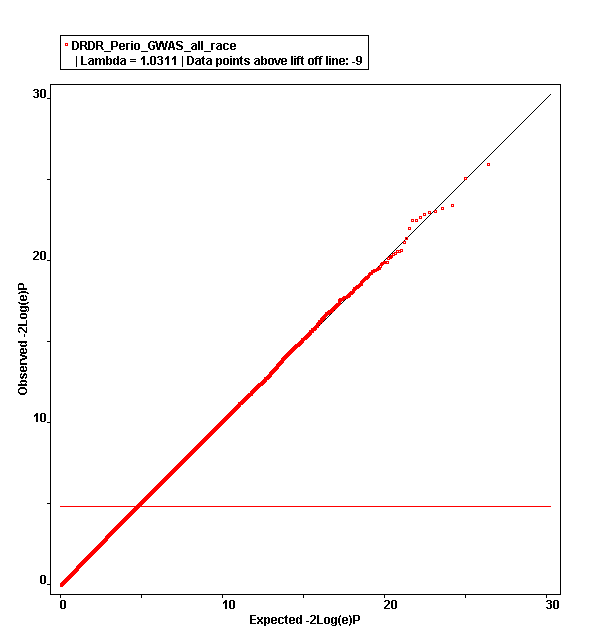

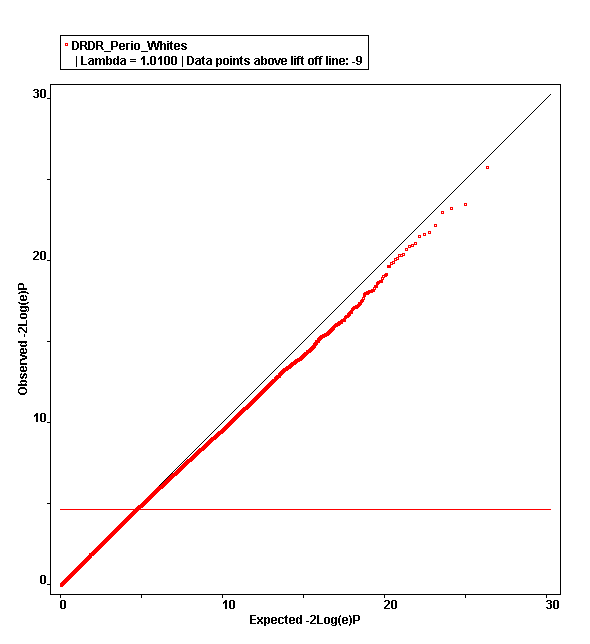


The Q-Q plots between the two scans show no important differences in lambda (genomic inflation factor).

**Appendix 5: Details of the Follow Up Samples**

The first consisted of a cohort from Porto Alegre. The Research Ethics Committee of the Federal University of Rio Grande do Sul, Porto Alegre, Brazil approved the study protocol. Subjects who agreed to participate signed a written informed consent form. The study sample included 1,460 DNA samples extracted from whole saliva. This population dataset has been described in detail elsewhere.^1,2^ Subjects with 30% or more teeth with clinical attachment loss of 5 millimeters or more were classified as having periodontitis. Four hundred and thirty individuals were defined as having chronic periodontitis and 1,030 individuals defined as unaffected. Individuals with a diagnosis of aggressive periodontitis were not included in the analysis. The mean age of affected individuals was 50.5 years (±12.5 years) and 202 were females and 228 were males. Ninety individuals were of African descent and 340 were White. Forty were diabetic and 255 smokers. Non-affected individuals had a mean age of 29.5 years (±12.5 years) and 582 were females and 448 males. One hundred and eighty-two individuals were of African descent and 848 were White. Nineteen were diabetic and 175 smokers (Table 1). With the exception of the ethnic and sex distributions, the frequency of the other demographic variables (diabetes status, smoking status, body mass index) was different between affected and unaffected individuals and strongly correlated with the age of the participants (data not shown).

The second cohort studied was from Rio de Janeiro. The Research Ethics Board at the Fluminense Federal University, Niterói, Rio de Janeiro, Brazil, approved this study. Informed consent was obtained from all participants. The study sample included 359 DNA samples extracted from whole saliva. Periodontal status was defined as described above and there were 183 individuals defined as having moderate to severe chronic periodontitis and 176 individuals defined as unaffected. The mean age of affected individuals was 58.3 years (±9.4 years) and 130 were females and 53 were males. Thirty-three individuals were of African descent and 150 were White. Twenty-five were diabetic and 39 smokers. Non-affected individuals had a mean age of 53.3 years (±11.9 years) and 127 were females and 49 males. Thirty-five individuals were of African descent and 141 were White. Six were diabetic and 13 smokers (Table 1). With the exception of the ethnic and sex distributions, the frequency of the other demographic variables (diabetes status and smoking status) was different between affected and unaffected individuals and strongly correlated with the age of the participants (data not shown).

**Results**

Supplemental Table 2: Top 100 hits from GWAS with all Samples


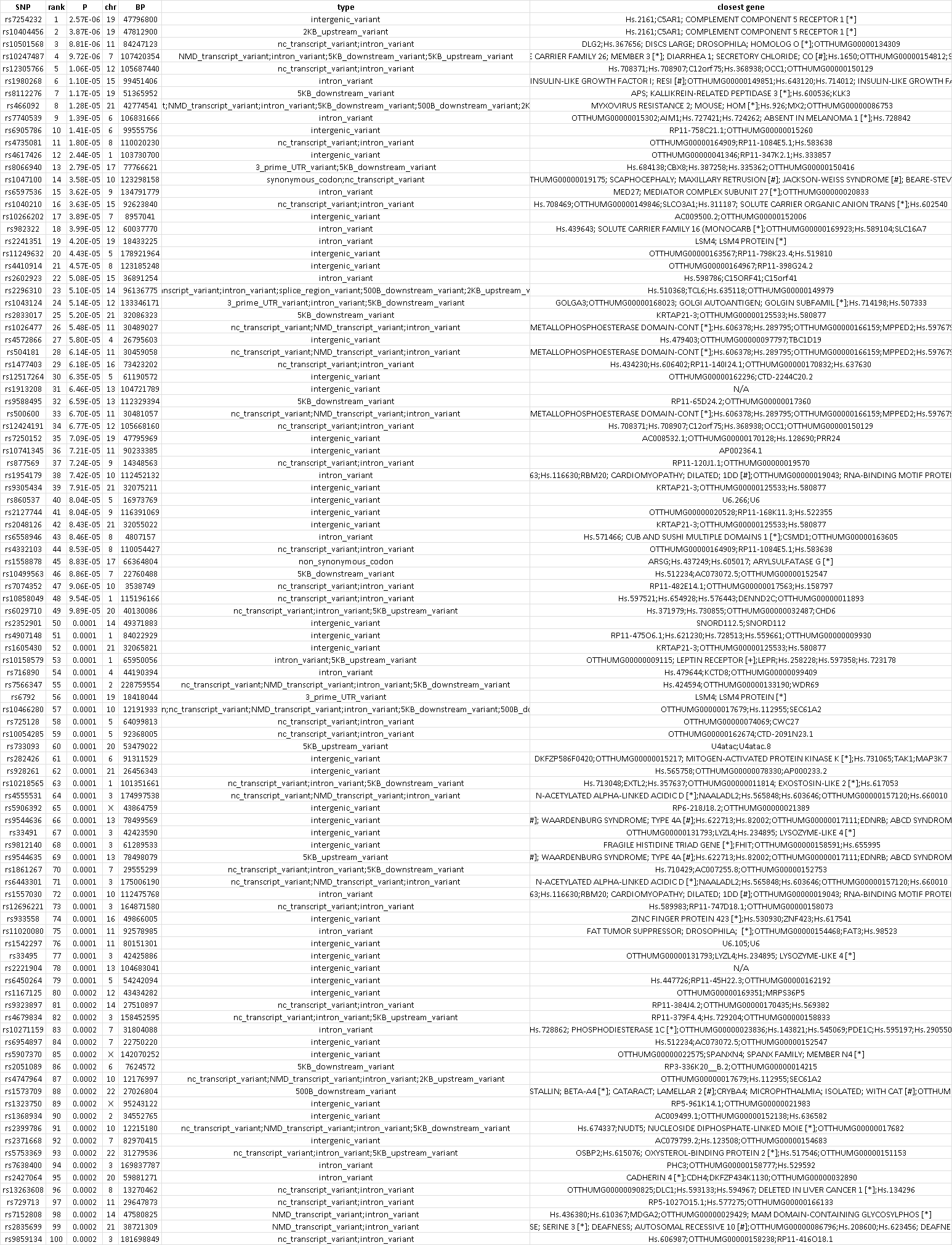


**Supplemental Table 3: Cross-comparison to Whites-only analysis (p**≤**0.001 in both Whites and all sample analyses)**


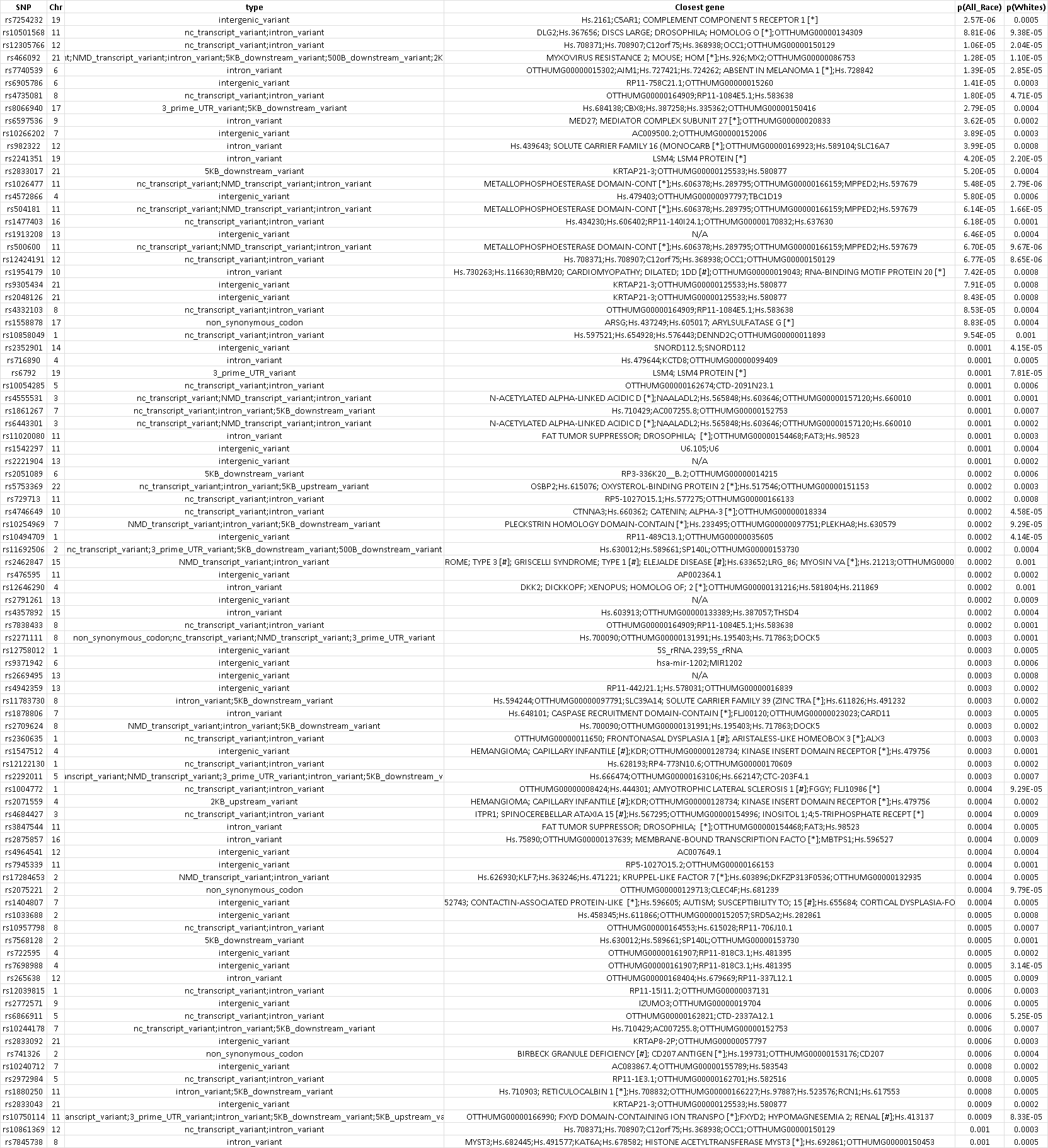

Supplement: Additional file 1 — Additional information on phenotype definition, power calculations and genome wide association analyses. [file 1472-6831-14-84-S1.doc]
